# Supplementary material for: Maternal Exposure to Cigarette Smoke during Pregnancy and Testicular Cancer in Offspring: A Systematic Review and Meta-Analysis
Source: Life (Basel). 2023 Feb 23;13(3):618. doi: 10.3390/life13030618 (PMC10054792; doi:10.3390/life13030618)

Figure S1. Forest plot of studies assessing maternal exposure to cigarette smoke during pregnancy and risk of seminoma testicular cancer in offspring [30,33,37,38]

Study	log[Odds Ratio]	SE	Weight	Odds Ratio IV, Random, 95% CI
McGlynn et al., 2006	-0.105	0.204	46.5%	0.90 [0.60, 1.34]
Pettersson et al., 2007	-0.713	0.321	19.6%	0.49 [0.26, 0.92]
Tuomisto et al., 2009	-0.598	0.776	3.4%	0.55 [0.12, 2.52]
Weir et al., 2000	-0.105	0.255	30.5%	0.90 [0.55, 1.48]
**Total (95% CI)**			**100.0%**	**0.79 [0.59, 1.04]**

Heterogeneity: $Tau^2 = 0.00$; $Chi^2 = 3.10$, $df = 3$ ($P = 0.38$); $I^2 = 3\%$
Test for overall effect: $Z = 1.67$ ($P = 0.09$)

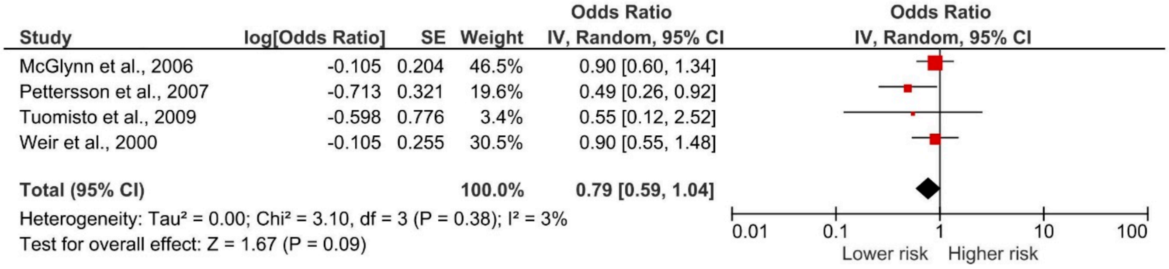

Supplement: Supplementary file 1 [file life-13-00618-s001.zip › Figure S1.pdf]
