# Supplementary material for: Maternal Exposure to Cigarette Smoke during Pregnancy and Testicular Cancer in Offspring: A Systematic Review and Meta-Analysis
Source: Life (Basel). 2023 Feb 23;13(3):618. doi: 10.3390/life13030618 (PMC10054792; doi:10.3390/life13030618)

Figure S2. Forest plot of studies assessing maternal exposure to cigarette smoke during pregnancy and risk of nonseminoma testicular cancer in offspring [30,33,37,38].

Study	log[Odds Ratio]	SE	Weight	Odds Ratio IV, Random, 95% CI
McGlynn et al., 2006	-0.105	0.204	44.1%	0.90 [0.60, 1.34]
Pettersson et al., 2007	0.166	0.301	20.5%	1.18 [0.65, 2.13]
Tuomisto et al., 2009	-0.342	0.286	22.7%	0.71 [0.41, 1.24]
Weir et al., 2000	0.405	0.383	12.7%	1.50 [0.71, 3.18]
**Total (95% CI)**			**100.0%**	**0.96 [0.74, 1.26]**

Heterogeneity: Tau² = 0.00; Chi² = 3.03, df = 3 (P = 0.39); I² = 1%

Test for overall effect: Z = 0.28 (P = 0.78)

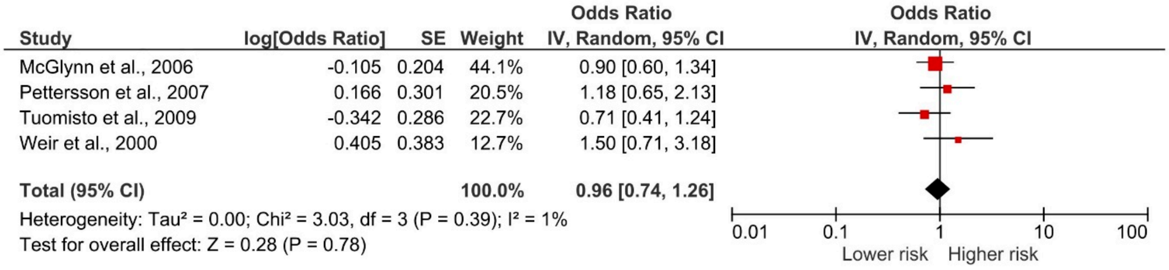

Supplement: Supplementary file 1 [file life-13-00618-s001.zip › Figure S2.pdf]
