# Supplementary material for: Maternal Exposure to Cigarette Smoke during Pregnancy and Testicular Cancer in Offspring: A Systematic Review and Meta-Analysis
Source: Life (Basel). 2023 Feb 23;13(3):618. doi: 10.3390/life13030618 (PMC10054792; doi:10.3390/life13030618)
Supplement: Supplementary file 1 [file life-13-00618-s001.zip › Table S1.pdf]

Table S1. Search protocol applied in the systematic literature search according to database and hits generated

Date	Database	Search string	Hits (n)
14/09/22	PubMed	**Block 1: Exposure**<br><br>"smoke"[MeSH Terms] OR "smoke"[All Fields] OR "smoke s"[All Fields] OR "smoked"[All Fields] OR "smokes"[All Fields] OR "Smoking"[MeSH Terms] OR "Smoking"[All Fields] OR "smokings"[All Fields] OR "smoking s"[All Fields] OR ("Nicotine"[MeSH Terms] OR "Nicotine"[All Fields] OR "nicotine s"[All Fields] OR "nicotines"[All Fields]) OR ("Cotinine"[MeSH Terms] OR "Cotinine"[All Fields] OR "cotinines"[All Fields]) OR "tobacco product*"[All Fields] OR ("Tobacco"[MeSH Terms] OR "Tobacco"[All Fields] OR "Tobacco Products"[MeSH Terms] OR ("Tobacco"[All Fields] AND "products"[All Fields]) OR "Tobacco Products"[All Fields] OR "tobaccos"[All Fields] OR "tobacco s"[All Fields]) OR "electronic nicotine delivery system*"[All Fields] OR "risk factor*"[All Fields] OR "cigarette smok*"[All Fields] OR ("vaped"[All Fields] OR "Vaping"[MeSH Terms] OR "Vaping"[All Fields] OR "vapes"[All Fields]) OR "Tobacco Smoke Pollution"[All Fields] OR ("Smoking"[MeSH Terms] OR "Nicotine"[MeSH Terms] OR "Cotinine"[MeSH Terms] OR "Tobacco Products"[MeSH Terms] OR "Tobacco"[MeSH Terms] OR "Electronic Nicotine Delivery Systems"[MeSH Terms] OR "Risk Factors"[MeSH Terms] OR "Cigarette Smoking"[MeSH Terms] OR "Vaping"[MeSH Terms] OR "Tobacco Smoke Pollution"[MeSH Terms]) OR "maternal smoking"[All Fields] OR "parental smoking"[All Fields] OR "paternal smoking"[All Fields] OR "electronic cigarette*"[All Fields] OR "tobacco snuff"[All Fields] OR ("tobacco, smokeless"[MeSH Terms] OR ("Tobacco"[All Fields] AND "smokeless"[All Fields]) OR "smokeless tobacco"[All Fields] OR "snuff"[All Fields] OR "snuffs"[All Fields]) OR ("tobacco, smokeless"[MeSH Terms] OR ("Tobacco"[All Fields] AND "smokeless"[All Fields]) OR "smokeless tobacco"[All Fields] OR "snus"[All Fields]) OR "nicotine gum"[All Fields] OR "nicotine patch*"[All Fields] OR ("smoking water pipes"[MeSH Terms] OR ("Smoking"[All Fields] AND "water"[All Fields] AND "pipes"[All Fields]) OR "smoking water pipes"[All Fields] OR "hookah"[All Fields] OR "hookahs"[All Fields])	1,632,345
		**Block 2: Outcome**<br><br>"Testicular Neoplasms"[MeSH Terms] OR "Germinoma"[MeSH Terms] OR "Seminoma"[MeSH Terms] OR "Nonseminomatous germ cell tumor"[Supplementary Concept] OR ("testicular neoplasm*"[All Fields] OR ("Germinoma"[MeSH Terms] OR "Germinoma"[All Fields] OR "germinomas"[All Fields]) OR ("Seminoma"[MeSH Terms] OR "Seminoma"[All Fields] OR "seminomas"[All Fields]) OR "Nonseminomatous germ cell tumor"[All Fields] OR "nonseminomatous germ cell tumour"[All Fields] OR "testis tumor"[All Fields] OR "testis tumour"[All Fields] OR "germ cell tumor"[All Fields] OR "germ cell tumour"[All Fields] OR ("nonseminomatous"[All Fields] AND ("Germinoma"[MeSH Terms] OR "Germinoma"[All Fields] OR "germinomas"[All Fields])) OR (("teste"[All Fields] OR "testi"[All Fields] OR "testis"[MeSH Terms] OR "testis"[All Fields] OR "testes"[All Fields] OR "inferior colliculi"[MeSH Terms] OR ("inferior"[All Fields] AND "colliculi"[All Fields]) OR "inferior colliculi"[All Fields]) AND ("nonseminoma"[All Fields] OR "nonseminomas"[All Fields]) AND ("cancer s"[All Fields] OR "cancerated"[All Fields] OR "canceration"[All Fields] OR "cancerization"[All Fields] OR "cancerized"[All Fields] OR "cancerous"[All Fields] OR "neoplasms"[MeSH Terms] OR "neoplasms"[All Fields] OR "cancer"[All Fields] OR "cancers"[All Fields])) OR ("testicular"[All Fields] AND ("nonseminoma"[All Fields] OR "nonseminomas"[All Fields]) AND ("cancer s"[All Fields] OR "cancerated"[All Fields] OR "canceration"[All Fields] OR "cancerization"[All Fields] OR "cancerized"[All Fields] OR "cancerous"[All Fields] OR "neoplasms"[MeSH Terms] OR "neoplasms"[All Fields] OR "cancer"[All Fields] OR "cancers"[All Fields])) OR "testis cancer"[All Fields] OR "testicular cancer"[All Fields] OR "testis carcinoma"[All Fields] OR "testicular carcinoma"[All Fields] OR "testis neoplasm*"[All Fields] OR "testicular tumor"[All Fields] OR "testicular tumour"[All Fields] OR ("nonseminoma"[All Fields] OR "nonseminomas"[All Fields]) OR "testis malignancy"[All Fields] OR "testicular malignancy"[All Fields] OR (("endocrinal"[All Fields] OR	56,450

		"endocrine system"[MeSH Terms] OR ("endocrine"[All Fields] AND "system"[All Fields]) OR "endocrine system"[All Fields] OR "endocrine"[All Fields] OR "endocrines"[All Fields] OR "endocrinic"[All Fields] OR "endocrinous"[All Fields]) AND ("male"[MeSH Terms] OR "male"[All Fields]) AND "cancer*"[All Fields]) OR "testis germ cell tumor"[All Fields] OR "testis germ cell tumour"[All Fields] OR "testicular germ cell tumor"[All Fields] OR "testicular germ cell tumour"[All Fields])	
		**Block 3: Window of exposure**<br><br>"Maternal Exposure"[MeSH Terms] OR "Prenatal Exposure Delayed Effects"[MeSH Terms] OR "maternal fetal exchange"[MeSH Terms] OR "Pregnancy"[MeSH Terms] OR "Fetus"[MeSH Terms] OR "maternal exposure*"[All Fields] OR "prenatal exposure delayed effect*"[All Fields] OR "maternal fetal exchange"[All Fields] OR "Pregnancy"[All Fields] OR "Fetus"[MeSH Terms] OR "Fetus"[All Fields] OR "fetuses"[All Fields] OR "fetus s"[All Fields] OR "foetu"[All Fields] OR "foetus"[All Fields] OR "Fetus"[MeSH Terms] OR "Fetus"[All Fields] OR "fetuses"[All Fields] OR "fetus s"[All Fields] OR "foetu"[All Fields] OR "foetus"[All Fields] OR "prenatal exposure*"[All Fields] OR "mother fetus relationship"[All Fields] OR "mother*"[All Fields] OR "pregnant woman"[All Fields] OR "pregnant women"[All Fields] OR "perinatal period"[All Fields] OR "prenatal period"[All Fields] OR "maternally"[All Fields] OR "maternities"[All Fields] OR "maternity"[All Fields] OR "mothers"[MeSH Terms] OR "mothers"[All Fields] OR "maternal"[All Fields] OR "in utero"[All Fields] OR "in utero"[All Fields] OR "fetale"[All Fields] OR "fetally"[All Fields] OR "fetals"[All Fields] OR "Fetus"[MeSH Terms] OR "Fetus"[All Fields] OR "fetal"[All Fields] OR "foetal"[All Fields] OR "fetale"[All Fields] OR "fetally"[All Fields] OR "fetals"[All Fields] OR "Fetus"[MeSH Terms] OR "Fetus"[All Fields] OR "fetal"[All Fields] OR "foetal"[All Fields] OR "gestation*"[All Fields] OR "perinatal"[All Fields] OR "perinatally"[All Fields] OR "perinatals"[All Fields] OR "peri-natal"[All Fields] OR "prenatal"[All Fields] OR "prenatally"[All Fields] OR "prenatals"[All Fields] OR "pre-natal"[All Fields]	1,631,421
		**Block 1 AND Block 2 AND Block 3**	368
14/09/22*	Embase®	**Block 1: Exposure**<br><br>smoking.mp. or smoking/<br>nicotine.mp. or nicotine/<br>cotinine.mp. or cotinine/<br>tobacco product*.mp.<br>tobacco/ or tobacco.mp.<br>electronic nicotine delivery system.mp.<br>risk factor/ or risk factor*.mp.<br>cigarette smoking.mp. or cigarette smoking/<br>vaping.mp. or vaping/<br>maternal smoking.mp. or maternal smoking/<br>parental smoking.mp. or parental smoking/<br>paternal smoking.mp. or paternal smoking/<br>electronic cigarette.mp. or electronic cigarette/<br>tobacco snuff.mp. or tobacco snuff/<br>snus.mp. or snus/<br>nicotine gum.mp. or nicotine gum/<br>nicotine patch.mp. or nicotine patch/<br>hookah.mp. or hookah/<br>passive smoking.mp. or passive smoking/<br>snuff.mp.<br>tobacco smoke pollution.mp.	2,117,797
		**Block 2: Outcome**<br><br>testicular neoplasm.mp.<br>germinoma.mp.<br>seminoma/ or seminoma.mp.<br>nonseminomatous germ cell tumor.mp.<br>nonseminomatous germ cell tumour.mp.<br>testis tumor.mp. or testis tumor/<br>testis tumour.mp.<br>germ cell tumor.mp. or germ cell tumor/	50,801

		nonseminomatous germinoma.mp. or non seminomatous germinoma/<br>testis nonseminoma cancer.mp. or testis nonseminoma cancer/<br>testicular nonseminoma cancer.mp.<br>testis cancer.mp. or testis cancer/<br>testicular cancer.mp.<br>testis carcinoma.mp. or testis carcinoma/<br>testicular carcinoma.mp.<br>testis neoplasm*.mp.<br>testicular tumor.mp.<br>nonseminoma.mp.<br>testis malignancy.mp.<br>testicular malignancy.mp.<br>endocrine male cancer.mp.<br>testis germ cell tumor.mp.<br>testis germ cell tumour.mp.<br>testicular tumour.mp.<br>testicular germ cell tumor.mp.<br>testicular germ cell tumour.mp.	
		**Block 3: Window of exposure**<br><br>maternal exposure.mp. or maternal exposure/<br>prenatal exposure delayed effects.mp.<br>maternal-fetal exchange.mp.<br>pregnancy.mp. or pregnancy/<br>fetus/ or fetus.mp.<br>foetus.mp.<br>prenatal exposure.mp. or prenatal exposure/<br>mother fetus relationship.mp. or mother fetus relationship/<br>mother.mp. or mother/<br>pregnant woman.mp. or pregnant woman/<br>pregnant women.mp.<br>perinatal period.mp. or perinatal period/<br>prenatal period.mp. or prenatal period/<br>maternal.mp.<br>in-utero.mp.<br>"in utero".mp.<br>fetal.mp.<br>foetal.mp.<br>gestational.mp.<br>perinatal.mp.<br>peri-natal.mp.<br>prenatal.mp.<br>pre-natal.mp.	1,689,035
		**Block 1 AND Block 2 AND Block 3**	381

/=EMTREE term
.mp. = Keyword term
*View full search string below (if you wish to run it, you have to copy it and paste into Ovid Search History Launcher - https://demo.ovid.com/demo/ovidsptools/launcher/launcher.html)
