# Supplementary material for: Maternal Exposure to Cigarette Smoke during Pregnancy and Testicular Cancer in Offspring: A Systematic Review and Meta-Analysis
Source: Life (Basel). 2023 Feb 23;13(3):618. doi: 10.3390/life13030618 (PMC10054792; doi:10.3390/life13030618)
Supplement: Supplementary file 1 [file life-13-00618-s001.zip › Table S2.pdf]

Table S2. Standardized form for the evaluation of completeness of reporting and risk of bias template

Systematic review: Maternal Exposure to Cigarette Smoking During Pregnancy and Testicular Cancer in Offspring: A Systematic Review and Meta-analysis.

Key-paper rating form for **[ADD ARTICLE NAME HERE]**:

A. Article and rater identification

B. Extraction of study characteristics

C. Completeness of reporting (adapted from Bonzini M *et al.* Occup Environ Med 2007)

D. Assessment of bias and confounding (adapted from Shamliyan TA *et al.* J Clin Epidemiol 2011 for specific needs for this review)

**A. Article and rater identification**	
Date for assessment	
Rater name	
First author, title, and publication year	
**Extraction of study characteristics**	
Location (country/region/area)	
Source population (general, defined by area, occupation, patients)	
Study design [cross-sectional, (nested) case control, cohort, ecological]	
Population size (exposed/unexposed; cases/referents)	
Exposure ascertainment [self-administered questionnaire, interview, biological samples (type if applicable: serum, cord blood, placenta, amniotic fluid, breast milk, urine, other)]	
Method chemical analysis (*if applicable,* biological samples)	
Outcome ascertainment (self-report, interview, examination, medical record, registry)	
Exposure contrast/exposure groups (e.g., maternal smoking vs non-smoking)	
Estimate, 95% CI. (Contact authors if not provided)	
Remarks (optional)	
**C. Completeness of reporting (is adequate information provided or not? No assessment of quality)**<br>Each issue is rated with 1 (adequate information) or 0 (not enough description), if 0, briefly indicate the main reason	
1. Study design	
2. Sampling frame and procedures	
3. Inclusion and exclusion criteria	

4. Population characteristics of exposed/unexposed or cases/referents	
5. Response rates reported or implicitly given	
6. Methods for exposure ascertainment	
7. Methods for outcome ascertainment	
8. Statistical analysis	
Completeness of reporting total score (0-8)	

D. Assessment of bias and confounding [sum range 0-16]

	Check	**High risk (2)** (likely risk of bias not addressed) * **Uncertain risk (1)** (information not provided) **Low risk (0)** (best practice) *If high risk: Justify your decision by short statements or quotes from the study
1. Reporting of tested hypotheses		High risk: E.g., incomplete/selective reporting of tested hypotheses compared to objectives or available data
		Uncertain risk
		Low risk: E.g., estimates presented for all hypotheses
2. Sample size justification (power calculations and/or addressing sample size in discussion)		High risk: E.g., small numbers may increase risk of false negative reporting
		Uncertain risk
		Low risk: E.g., justification provided by authors
3. Selection bias cohort studies (attrition)/ case-control studies (non-response)		High risk: E.g., loss to follow-up larger than 20% or differ more than 10% between exposed and unexposed
		Uncertain risk: E.g., not reported
		Low risk: E.g., loss to follow-up less than 20% with no difference between groups
4. Information bias (exposure ascertainment)		High risk: E.g., exposure identified by patient recall in questionnaires or interviews
		Uncertain risk: E.g., methods not described
		Low risk: E.g., exposure identified by medical examination/record or from validated registries
5. Information bias (outcome ascertainment)		High risk: E.g., outcome identified by patient recall in questionnaires or interviews
		Uncertain risk: E.g., methods not described
		Low risk: E.g., outcome identified by medical examination/record or from validated registries
6. Confounding (E.g., Are relevant factors considered? Judgement specific for outcome).		High risk: E.g., no confounding considered. Major confounding factors/effect modifiers (Mothers: maternal age, parity, SES, ethnicity, gestational age, pre-pregnancy BMI. Father: testicular cancer, age ethnicity. Sons: age at diagnosis).

		Uncertain risk: E.g., adjustment for confounding factors not reported
		Low risk: E.g., major confounding factors/effect modifiers adequately accounted for by design and/or analysis
7. Measuring of confounding factors		High risk: E.g., non-valid or inadequate reporting or measures of confounding factors
		Uncertain risk: E.g., source and methods for collection of data not reported
		Low risk: E.g., adequate, and non-differential ascertainment of information
8. Exposure-response		High risk: E.g., exposure-response not addressed in the study
		Uncertain risk
		Low risk: E.g., exposure-response adequately addressed in the study
Bias and confounding total score (0-16)		
